# Multi-centre evaluation of Gram stain in the diagnosis of septic arthritis

**DOI:** 10.5194/jbji-10-61-2025

**Published:** 2025-03-17

**Authors:** Charlotte Smith, Robert J. Maloney, Deborah Wearmouth, Hemant Sharma, Kordo Saeed, Nusreen Ahmad-Saeed, Rachel Annett, Lucinda Barrett, Sara E. Boyd, Peter Davies, Harriet Hughes, Gwennan Jones, Laura Leach, Maureen Lynch, Deepa Nayar, Martin Marsh, Shanine Mitchell, Lynn Moffat, Luke S. P. Moore, Michael E. Murphy, Shaan Ashk O'Shea, Teresa Peach, Christina Petridou, Niamh Reidy, Ben Talbot, Catherine Aldridge, Gavin Barlow

**Affiliations:** 1 Department of Infection, University Hospital Southampton NHS Foundation Trust, Southampton, UK; 2 School of Clinical and Experimental Sciences, University of Southampton, Southampton, UK; 3 Public Health Wales, Hot Lab University Hospital of Llandough, Cardiff, Wales, UK; 4 Department of Infection, Hull University Teaching Hospitals NHS Trust, Hull, UK; 5 Experimental Medicine and Biomedicine, York Biomedical Research Institute, Hull York Medical School, University of York, York, UK; 6 Oxford University Hospitals (OUH), Oxford, UK; 7 David Price Evans Global Health and Infectious Disease Research Group, University of Liverpool, Liverpool, L69 3GE, UK; 8 National Institute for Health Research Health Protection Research Unit in Healthcare Associated Infections and Antimicrobial Resistance, Imperial College London, Du Cane Road, London, W12 0HS, UK; 9 Infection Clinical Academic Group, St. George's Hospital NHS Foundation Trust, Blackshaw Road, London, SW17 0QT, UK; 10 Chelsea and Westminster NHS Foundation Trust, London, UK; 11 Imperial College Healthcare NHS Trust, North West London Pathology, Fulham Palace Road, London, UK; 12 Department of Clinical Microbiology, Mater Misericordiae University Hospital, Dublin, Ireland; 13 Department of Microbiology, NHS Greater Glasgow and Clyde, Glasgow Royal Infirmary, New Lister Building, Alexandra Parade, Glasgow, UK; 14 Public Health Wales Department of Microbiology, University Hospital of Wales, Cardiff, Wales, UK; 15 Department of Microbiology, Newcastle upon Tyne Hospitals NHS Foundation Trust, Newcastle upon Tyne, UK; 16 Department of Orthopaedics, Newcastle upon Tyne Hospitals NHS Foundation Trust, Newcastle upon Tyne, UK; 17 College of Medical, Veterinary & Life Sciences, University of Glasgow, Wolfson Medical School Building, Glasgow, UK; 18 Public Health Wales, Health Protection and Infection Division, Capital Quarter, Cardiff, Wales, UK; 19 Department of Infection, Hampshire Hospitals NHS Foundation Trust, Winchester, UK; 20 Department of Trauma and Orthopaedics, Hull University Teaching Hospitals NHS Trust, Hull, UK; 21 Sheffield Health & Social Care NHS Foundation Trust, Centre Court, Atlas Way, Sheffield, S4 7QQ, UK

## Abstract

**Introduction**: Gram stain of synovial fluid is a rapid test for the diagnosis of native joint septic arthritis. Single-centre studies have suggested Gram stain will miss a considerable proportion of patients who are subsequently synovial-fluid-culture-positive or polymerase chain reaction (PCR)-positive. The object of this study was to reassess Gram stain in a large, multi-centre cohort of patients from the United Kingdom (UK) and Ireland. **Methods**: The study was a retrospective analysis combining two large datasets. We defined septic arthritis microbiologically as at least one positive joint aspirate culture and/or PCR test. “Best case” and “worst case” definitions were applied depending on the likelihood organisms were true infecting pathogens. **Results**: Gram stain missed a high proportion of culture-/PCR-positive patients using both the best (74 % missed) and worst (81 % missed) case definitions. Using the best case definition, the sensitivity of Gram stain was 0.26, specificity 0.99, positive predictive value 0.84, negative predictive value 0.87, accuracy 0.87, and area under the receiver operator curve 0.62 (95 % CI 0.57 to 0.68, p<0.001 ). False positive Gram stains were infrequent (1 %). Age, joint involved, and other synovial fluid characteristics were less predictive of a positive culture/PCR than Gram stain. **Conclusions**: While a positive synovial fluid Gram stain should always be considered to indicate potential septic arthritis, a negative Gram stain, regardless of synovial fluid crystals or white cell count, should not be used to rule out septic arthritis. The value of Gram stain as an urgent out-of-hours test for septic arthritis is open to considerable debate.

## 1 Introduction

Septic arthritis of a native large joint commonly presents with non-specific features mimicking other acute joint conditions (Coakley et al., 2006). The incidence in the UK between 1998 and 2013 increased by 43 %, with an incidence of 7.8 per 100 000 by 2013 (Rutherford et al., 2016). It is crucial to have a timely, clear diagnosis as delayed management may result in sub-optimal treatment, joint damage, and even mortality (McBride et al., 2020). Long-term morbidity affects up to one-half of patients, with the need for joint replacement or other surgeries in some (Lauper et al., 2018; McBride et al., 2020).

The diagnosis of native septic arthritis is based on synthesizing the clinical presentation, radiological, and blood test findings alongside joint aspiration results. A microbiological diagnosis is achievable in approximately 70 % to 80 % of cases based on joint aspirate fluid or operative specimen culture (Cunningham et al., 2014; Holzmeister et al., 2021). Newer technologies, such as polymerase chain reaction (PCR), increase yield (Saeed et al., 2023). When one of these tests is positive for a recognized microbiological cause, it is diagnostic of septic arthritis. While most causative bacteria are Gram-positive, Gram-negatives occur in up to one of five cases, so an early microbiological diagnosis is vital to guide antibiotics (Arieli et al., 2021; McBride et al., 2020). In the UK and Ireland, the standard-of-care empiric treatment is intravenous (IV) flucloxacillin (or equivalent agent), which covers susceptible Gram-positive bacteria only (Coakley et al., 2006).

A synovial fluid Gram stain and culture are the “gold standard” microbiological tests (UK SMIs, 2023). The Gram stain is often performed rapidly and is generally considered to be an indicator of the likelihood of infection. Not all laboratories offer a 24 h service, meaning biomedical scientists may have to come in out of hours. Anecdotally, a negative Gram stain or the aspirate being positive for crystals is often considered an indication that infection is unlikely and can result in patient discharge. This can result in patient recall and is supported by previous studies that showed a high proportion of false negatives (e.g. 78 %) (Stirling et al., 2014). Given concerns about Gram stain in septic arthritis, the object of this study was to reassess its performance in a large multi-centre study in the UK and Ireland, where microbiological investigation is standardized by national standards (UK SMIs, 2023).

## 2 Methods

The study was a retrospective analysis involving eight large teaching hospitals in the UK and Ireland. Two datasets were combined, one from a multi-centre study of the BioFire PCR native joint infection panel (Saeed et al., 2023) and an unpublished dataset from a 1200-bed UK teaching hospital (no duplication in the two cohorts).

The national dataset was collected over 6 months at each site between March 2021 and March 2022. The single-centre dataset was collected during 26 June 2019 to 26 June 2021 (both continuous). All patients had a joint aspirate from a large native joint followed by microbiological assessment based on the UK's Standards for Microbiology Investigations (SMI) (UK SMIs, 2023). Prosthetic joints were excluded.

**Table 1:** Definitions of septic arthritis according to organism identified.

Best case definition (i.e. more likely to be true causal pathogens): *Staphylococcus aureus*,
*Enterococcus faecalis*, *Enterococcus faecium*, Group A and B and C streptococci, *Pseudomonas* spp.,
*Kingella kingae*, *Neisseria gonorrhoea*, *Proteus mirabilis/vulgaris*, *Escherichia coli*, alpha haemolytic
streptococci including *Streptococcus pneumoniae*, *Haemophilus influenzae/parainfluenzae*, *Klebsiella*
*pneumoniae/oxytoca*, *Enterobacter cloacae* complex, *Serratia marcescens*, *Morganella morganii*,
*Citrobacter freundii*, *Providencia rettgeri*, *Candida albicans*, *Bacteroides fragilis*,
*Clostridium perfringens*, and *Fusobacterium* spp.
Worst case definition (i.e. the above best case organisms and the following as less likely
pathogens): Corynebacterium spp., *Bacillus* spp., *Enterococcus raffinosus*, *Cutibacterium acnes*,
*Enterococcus mundtii*, *Enterococcus casseliflavus*, *Paenibacillus* spp., non-albicans *Candida* spp.,
*Dermacoccus nishinomiyaensis*, *Moraxella osloensis*, Coagulase-negative staphylococci
(CoNegS), *Anaerococcus* spp., *Finegoldia magna*, *Parvimonas micra*, *Peptoniphilus* spp., and
*Peptostreptococcus anaerobius*

Data were audited and integrated into an Excel for Windows spreadsheet. Age and synovial fluid white cell count (WCC) were collected differently in the two studies such that when combined, patients were grouped into <18 , 19–55, or >55 years for age and <10 and >10 white cells per high-powered field (HPF) for synovial fluid WCC. We did not collect patient data other than age and joint involved.

We defined septic arthritis microbiologically based on the presence of at least one positive direct or enrichment aspirate culture (when performed) and/or positive PCR test. Some organisms were considered potential contaminants rather than true pathogens, so we created two definitions: a “best case” definition based on organisms very likely to be true pathogens and a “worst case” definition based on best case organisms and those with a higher risk of being contaminants according to literature review and authors' experience (Table 1).

### 2.1 Statistical analyses

SPSS version 28 was used for statistical analyses. Descriptive statistics are given as medians or percentages with 95 % confidence intervals (CIs) where appropriate. Sensitivity, specificity, positive predictive value (PPV), negative predictive value (NPV), accuracy, the area under the receiver operator curve (AUROC), and associated 95 % CIs were calculated. The definitions of performance characteristics used are given in Table A1. Statistical associations between synovial fluid characteristics, the joint involved, and age were analysed using $\chi^{2}$ and multivariate binary logistic regression with a positive culture/PCR result (using both definitions in different models) as the dependent variable.

Only predictor variables with a p≤0.1 on univariate analyses were included in multivariate analyses. Logistic regression models were constructed with adjusted odds ratios, 95 % CIs, and p values from models considered the most clinically and statistically robust presented. P values < 0.05 were considered to indicate statistical significance. Analyses were performed by the total number of samples and by patient with results for both presented. When analysed by patient, only one sample was considered, either their index (first) sample if all others were negative by culture/PCR or the positive sample if the first sample was negative or the “most” positive sample if more than one sample was positive (e.g. if two organisms were identified in the first sample and only one in a second, the first sample was used).

## 3 Results

Overall, our results, and conclusions thereof, were very similar regardless of whether analyses were by sample or patient or using the best case or worst case definitions of septic arthritis. Here we present the results by sample, and patient as appropriate, using the best case definition. To allow comparison, results by patient using the worst case definition are presented in the Appendix. The inclusion or exclusion of PCR results as part of our definitions did not change the results or conclusions thereof.

**Figure 1:**
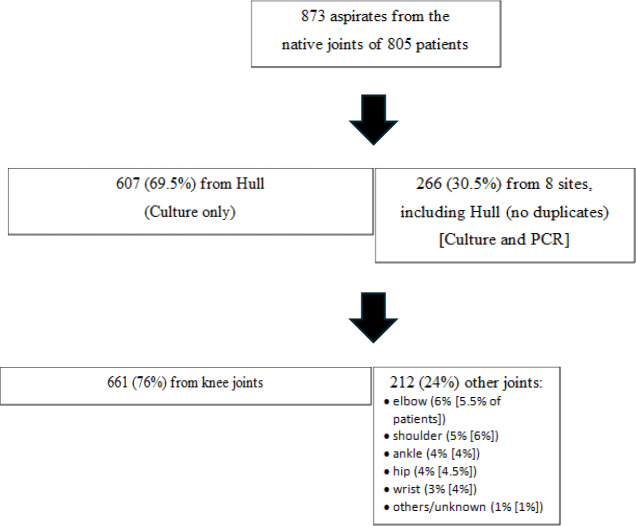
Number and nature of the joint aspirates included.

The number and characteristics of joint aspirates are in Fig. 1. The patient's exact age was only collected in the single centre study, with the mean age being 64 years (median = 67, range 2 to 99 years). Overall, 69.5 % ( N=557 /801) were >55 years old (65 % and 72 % in the two cohorts). Only 2.5 % of patients were <18 years old. A Gram stain was available for 98 % of samples ( N=856 ), with 95 % being negative ( N=812 ) and the same proportion negative in the two cohorts (95 %).

### 3.1 Synovial fluid culture and PCR, crystals, and white cell count

N=211 samples (24 %) had a positive direct or enrichment synovial fluid culture (also 24 % in those with a Gram result; 21 % at the largest site versus 31 % at other sites); 26 % of samples were positive by either culture and/or PCR. Of patients with at least one positive sample by culture/PCR, most were considered to have septic arthritis using the best case definition ( N=136 , 65 %); 129/201 (64 %) in those with a Gram stain result.

Crystals were common: 25 % ( N=218/860 ) of samples overall with 32 % ( N=191/594 ) at the centre contributing most and 10 % ( N=27 /266) at other centres. One in nine patients (11 %) with positive crystals had septic arthritis by our best case definition. Of all samples, 3 % ( 27/860 ) had both positive crystals and a positive culture/PCR test by the best case definition.

Of all samples, 40 % ( 352/873 ) had a synovial fluid white cell count > 10 per HPF (54 % < 10, 6 % unknown/not done; 31 % and 46 % > 10 per HPF in the two datasets). Of patients with a WCC > 10 per HPF ( N=308 ), 27 % were deemed to reflect septic arthritis by the best case definition versus 10 % in those with a WCC < 10 ( N=446 ).

**Table 2:** Two by two table for calculation of performance characteristics by sample and using the best case microbiological definition for septic arthritis.

Culture-	Gram	Gram	Totals
or PCR	stain	stain	
positive	positive	negative	
(i.e. septic			
arthritis)			
Yes	37	107	144
No	7	705	712
Totals	44	812	856

### 3.2 Relationship between Gram stain and culture/PCR

Of samples with a positive Gram stain ( N=44 ), culture or PCR was positive in 84 % ( N=37) using the best case definition. Of samples with a negative Gram stain ( N=812) , culture/PCR was positive in 107 (13 %) using the best case definition.

Of samples with a positive culture/PCR ( N=144 ) using the best case definition, the Gram stain was positive in 26 % ( N=37 ). Of samples with a negative culture/PCR ( N=712 ), the Gram stain was positive in 1 % ( N=7 ) (false positives) using the best case definition.

**Table 3:** Univariate and multivariate analyses.

Patient characteristic	Percentage with	$\chi^{2}$ and	Multivariate p value,
	septic	univariate	odds ratio (OR), and
	arthritis	p values	95 % confidence
	(best case		intervals
	definition)		
56 years old or more	15 %	3.8, p=0.05	OR 0.5, 95 % CI 0.31
			to 0.79, p=0.003
			(older age reduced
			risk)
<56 years old	20.5 %		
Synovial fluid white cell	27 %	38, p<0.001	OR 3.5, 95 % CI 2.2 to
count > >10 per HPF			5.6, p<0.001
Synovial fluid white cell	10 %		
count < 10 per HPF			
Synovial fluid Gram-stain-	85 %	150, p<0.001	OR 32.9, 95 % CI 12.5
positive			to 86.2, p<0.001
Synovial fluid Gram-stain-	13 %		
negative			
Synovial fluid crystals	11 %	5.1, p=0.024	OR 0.38, 95 % CI 0.2
positive			to 0.69, p=0.002
			(crystals reduced risk)
Synovial fluid crystals	18 %		
negative			
Affected joint *was* the knee	14 %	11.2, p<0.001	Not significant
			( p=0.079 )
Affected joint *was not* the knee	25 %		

Of patients who had a Gram-positive organism cultured or by PCR (best case definition), 24.5 % had a positive Gram stain test versus 10.5 % of patients with Gram-negative organisms and 12.5 % in those with both a Gram-positive and a Gram-negative organism ( $\chi^{2}=2.35$ , P>0.3 ).

### 3.3 Performance characteristics

Using the best case definition by sample, the sensitivity of Gram stain was 0.26, specificity 0.99, positive predictive value (PPV) 0.84, negative predictive value (NPV) 0.87, and accuracy 0.87; see Table 2. By patient, the same performance characteristics were 0.27, 0.99, 0.85, 0.87, and 0.87, respectively. The AUROC was 0.62 (95 % CI 0.57 to 0.68, p<0.001 ) using the best case definition.

### 3.4 Univariate and multivariate analyses of Gram stain by patient

A summary of analyses is in Table 3. In multivariate analyses (binary logistic regression) with septic arthritis, using the best case definition, as the dependent variable and variables identified in univariate analyses as potential predictors, older age and crystals but not the joint being the knee, were found to be associated with a reduced likelihood of septic arthritis. In contrast, a synovial fluid WCC ≥ 10 per HPF and Gram stain positivity were associated with an increased risk of septic arthritis: Hosmer–Lemeshow test $\chi^{2}=6.4$ , p=0.49 . There was a statistically significant association ( $\chi^{2}=6.6$ , p=0.01 ) between older age and whether the joint aspirate was from the knee. Removing one or other or both from the model improved the model's statistical performance (Hosmer–Lemeshow test) while strengthening the lack of association between the joint involved and septic arthritis when this variable was retained. This did not change our overall results or conclusions.

### 3.5 Microbiology

The most identified bacteria are in Table 4. Approximately 1 in 15 patients (6.5 %) with a positive culture/PCR had >1 organism identified. The proportion of patients with a positive Gram stain was much higher in patients with septic arthritis according to the best case definition versus patients with organisms deemed less likely to represent true septic arthritis (27 % (best case) versus 4 % (worse case) versus 0.5 % (culture-negative), $\chi^{2}=152$ , P<0.001 ). A similar association was identified for high synovial fluid WCC ( ≥10 per HPF) (66 % (best case) versus 28 % (worse case) versus 37 % (culture-negative), $\chi^{2}=40$ , P<0.001 ).

**Table 4:** Bacteria identified by culture or PCR (i.e. all organisms identified regardless of whether best or worst case organisms).

Bacteria	Number of	Percentage of all
	patients	patients
	with this	with a
	organism	positive
	on culture	culture
	or PCR	or PCR
		( N=201 )
Methicillin-sensitive *Staph. aureus*	74	37 %
Gram-negative bacteria (excluding	23	11 %
*Pseudomonas* and *Neisseria*		
species)^*^		
Coagulase-negative staphylococci	23	11 %
Other streptococci (unidentified or	18	9 %
viridans group)		
Other bacteria	16	8 %
Lancefield Group A, B, and G	13	6 %
streptococci		
Enterococci species	10	5 %
Methicillin-resistant *Staph. aureus*	6	3 %
*Streptococcus pneumoniae*	6	3 %
*Pseudomonas* species	5	2.5 %
*Neisseria gonorrhoeae*	3	1.5 %

^*^ Gram-negative bacteria: *Citrobacter* species (0.5 %) *Escherichia coli* (3.5 %), *Enterobacter* species (0.5 %), *Haemophilus parainfluenzae* (1.5 %), *Kingella kingae* (0.5 %), *Klebsiella pneumoniae* (1.5 %), *Moraxella* species (0.5 %), *Proteus* species (2 %), *Providencia* species (0.5 %), and *Serratia* species (0.5 %).

## 4 Discussion

Our key finding is that a negative synovial fluid Gram stain, regardless of other sample characteristics or patient age or joint involved, or the nature of the microbiological definition, cannot rule out septic arthritis. Indeed, Gram stain will miss most (74 %) subsequently culture-/PCR-positive synovial fluid specimens. In contrast, the vast majority of patients with a positive Gram stain will have a positive culture/PCR; false positives are rare. The accuracy of Gram stain and culture/PCR can depend on factors such as operator experience or error and patient exposure to antibiotics, although such factors reflect the realities of real life. While a positive Gram stain is undoubtedly useful, in our experience it tends to be negative Gram stains, particularly when crystals are present, that are more likely to lead to poor clinical decisions and under-intervention.

A summary of the performance characteristics of Gram stain for the diagnosis of native joint septic arthritis based on large ( N≥100 patients) published studies, identified during a PubMed search using the keywords *septic*, *infective*, *infectious*, *arthritis*, *arthropathy*, *synovial*, and *Gram stain*, is in Table 5 (studies that mixed native and prosthetic joint data when they could not be extracted separately were excluded). We identified five studies in addition to this study (3051 patients). Results were similar across studies with sensitivity ranging from 0.17 to 0.4, specificity 0.97 to 0.99, PPV 0.84 to 0.99, NPV 0.38 to 0.9, and accuracy 0.56 to 0.9. False negatives were high in all studies (60 % to 83 %; 73 % to 83 % when the one paediatric study is excluded). Given the large number of patients across studies and consistency of findings, we have a high level of confidence in our conclusions. All studies were performed in high-income settings.

**Table 5:** A summary of published studies to date (including this study).

Study	Sensitivity	Specificity	PPV	NPV	Accuracy	AUROC	Other results/
Number of							comments
patients/							
samples							
(Reference)							
Smith et al. (2025)	0.26	0.99	0.84	0.87	0.87	0.62	False negatives =
N=805	$\left[0.19^{*}\right]$	$\left[0.99^{*}\right]$	$\left[0.93^{*}\right]$	$\left[0.78^{*}\right]$	$\left[0.79^{*}\right]$	$\left[0.59^{*}\right]$	74 %
UK							
(this study;							
multi-centre)							
Al-Tawil et	0.22	0.99	0.87	0.9	0.9	–	16 % prosthetic
al. (2021)							joints
N=698							False negatives =
UK							78 %
(single centre)							
Bram et al.	0.4	0.97	0.87	0.76	0.78	–	Paediatric only
(2018)							False negatives =
N=302							60 %
USA							
(single centre)							
Stirling et al.	–	–	–	–	–	–	False negatives =
(2014)							78 %
N=143							
UK							
(single centre)							
Gbejuade et	0.17	0.99	0.89	0.89	0.9	–	False negatives =
al. (2019)							83 %
N=830							
samples							
UK							
(single centre)							
Cunningham	0.40	0.99	0.99	0.38	0.56	–	False negatives =
et al. (2014)							73 %
N=273							
Switzerland							
(single centre)							

In keeping with others (Prior-Español et al., 2019), our results show synovial fluid with both crystals and positive culture/PCR is relatively common. Crystals should not rule out septic arthritis, even though this was predictive in multivariate analyses. Likewise, synovial fluid WCC at a cut-off of ≥10 per HPF was much less predictive of septic arthritis than Gram stain. In their systematic review, Walinga et al. (2021) found a synovial fluid WCC with a cut-off of 50 000 mm^−3^ was the most applied in studies with a sensitivity from 53 % to 100 % and specificity 66 % to 97 %. The proportion of synovial polymorphonuclear cells with cut-offs ranging from 75 % to 95 % showed a sensitivity from 42 % to 100 % and specificity 54 % to 94 %. Holzmeister et al. (2021) developed a calculator using 281 patients to help in the diagnosis of knee septic arthritis. Synovial WCC ≥ 30 000 µ L had the highest odds ratio (91) compared to other statistically significant predictors: Gram stain positivity (21.5), duration of pain > 2 d (7), prior septic arthritis (5), knee effusion (5), and synovial fluid crystals (0.1) (Holzmeister et al., 2021). As far as we know, this calculator has not been validated in other large cohorts or joints.

We found older age significantly less associated with septic arthritis. This is likely to be due to a higher proportion of older adults presenting with non-infective causes of an acute joint. The joint involved (knee versus others) was not useful in predicting subsequent culture/PCR result. *Staphylococcus aureus* was the most common organism identified in patients with a positive culture/PCR, with more than three-quarters of culture-/PCR-positive patients having Gram-positive bacteria. It is worth noting that 15 % of patients with a positive culture/PCR (all organisms; 23 % in those with a best case organism) had Gram-negative bacteria, in keeping with the published literature (McBride et al., 2020) and highlighting the importance of identifying patient risk factors for septic arthritis caused by Gram-negative bacteria to guide empiric antibiotic therapy. Approximately double the proportion of patients had a positive Gram stain when only Gram-positive bacteria were identified, although this was not statistically significant.

Walinga et al. (2021) also found serum erythrocyte sedimentation rate (ESR), C-reactive protein (CRP), procalcitonin, and synovial fluid Gram stain to be useful tests for septic arthritis, although all had variable performance, and the quality and the size of studies were generally low; this study is larger than the largest study in that review. Gram stain ( N=5 studies) had a sensitivity and specificity ranging from 27 % to 81 % and 99 % to 100 %, respectively. Dey et al. (2023) performed a systematic review of the diagnosis of the acute joint but were unable to meta-analyse studies that had assessed Gram stain. Of other synovial fluid markers, leucocyte esterase had the highest pooled sensitivity (0.94 (95 % 0.70 to 0.99); specificity 0.74 (0.67 to 0.81); AUROC 0.78), while lactate ( ≥10 mmol L^−1^) had the highest pooled specificity (0.99 (0.96 to 1.0); sensitivity 0.36 (0.22 to 0.53); AUROC 0.85), and both tumour necrosis factor- α (36 pg mL^−1^) and procalcitonin (0.5 µ g L^−1^) had the highest pooled AUROCs (0.93; TNF α sensitivity 0.86 (0.49 to 0.97), specificity 0.88 (0.54 to 0.98) and procalcitonin sensitivity 0.67 (0.26 to 0.92), specificity 0.93 (0.84 to 0.97)). Emerging technologies that could be used instead of Gram stain in the rapid diagnosis of septic arthritis need further evaluation.

### Limitations

As with all observational studies, there is a risk of bias and confounding. Although the two amalgamated cohorts were similar, some characteristics were different, which likely reflects factors such as study design, local clinical practices, and differences in patient populations. Although we used pragmatic microbiological definitions, most published studies to date have done similarly, and there is no accepted “gold standard” definition of septic arthritis. It is also important to acknowledge that culture-/PCR-negative septic arthritis is well recognized.

Likewise, we used best and worst case definitions to reflect that some results may be due to specimen contamination. We acknowledge that by classifying all coagulase-negative staphylococci (CoNegS) in the worst case definition, organisms such as *Staphylococcus lugdunensis* that are more likely to be pathogens may have been misclassified. Not all laboratories in this study identified CoNegS to species level, but of those that did only *Staphylococcus capitis* (1 % of all organisms identified), *Staphylococcus epidermidis* (0.5 %), *Staphylococcus hominis* (0.5 %), and *Staphylococcus warneri* (0.5 %) were identified. Some may argue that all positive synovial fluid culture or PCR results are clinically significant. Without the context of detailed clinical data, we cannot confirm or refute this, but our approach is supported by a much higher proportion of worst case organisms growing on enrichment only (82 %), which is more likely to be associated with contamination, versus best case organisms (24 %). Despite these limitations, this is the largest such multi-centre study to date, performed within health systems with national standards for microbiological investigations and that is consistent with, supports, and adds to previous research.

## 5 Summary/conclusion

In this large multi-centre study, Gram stain missed most patients with a synovial fluid aspirate subsequently positive by culture or PCR. In keeping with previous work, a negative Gram stain, regardless of other synovial fluid characteristics, the joint involved, or patient age, does not rule out septic arthritis.

## Data Availability

The data are currently being used for ongoing projects – however, requests to access the data are welcomed and can be done by sending an e-mail to gavin.barlow@york.ac.uk.

## Appendix A

### A1 Additional results presented by patient and using the worst case definition as appropriate

A Gram stain was available for 98 % of patients ( 788/805 ), with 93 % being negative ( 747/788 ).

### A2 Synovial fluid culture and PCR, crystals, and white cell count

In total, 24 % of patients had a positive direct or enrichment synovial fluid culture. Of patients with at least one positive sample by culture or PCR, most (64 %) were considered to have septic arthritis using the worst case definition ( 129/201 ) in those with a Gram stain result.

Crystals were identified in 24 % ( N=193/793 ) of patients, with 31.5 % at the centre contributing most and 10 % at other centres. One in six patients (17 %) with positive crystals had septic arthritis by our worst case definition; 4.5 % ( 39/860 ) of samples had both positive crystals and a positive culture/PCR test by the worst case definition, which is 4 % of patients ( 33/793 ).

Of patients with a WCC > 10 per HPF ( N=308) , 33 % were deemed to reflect septic arthritis by the worst case definition versus 21 % in those with a WCC < 10 ( N=446 ).

### A3 Relationship between Gram stain and culture/PCR

Of patients with a positive Gram stain ( N=41 ), culture or PCR was positive in 85 % ( N=35 ) using the best case definition and 93 % of samples and patients using the worst case definition. Of patients with a negative Gram stain, culture/PCR was positive in 94 (13 %) using the best case definition (22 % ( 163/747 ) using the worst case definition).

Of samples with a positive culture/PCR ( N=218 ) using the worst case definition, Gram stain was positive in 19 % ( N=41 ); 35/129 (27 %) and 38/201 (19 %) of patients using the best case and worst case definitions, respectively. Of samples with a negative culture/PCR ( N=712 ), Gram stain was positive in 0.5 % ( 3/638 ) (false positives) using the worst case definition and 6/659 (1 %) and 3/587 (0.5 %) of patients using the best case and worst case definitions, respectively.

Of patients who had a Gram-positive organism cultured or identified by PCR (worst case organisms), 31 % had a positive Gram stain test versus 12.5 % of patients with Gram-negative organisms and 12.5 % in those with both a Gram-positive and a Gram-negative organism ( $\chi^{2}=3.3$ , P>0.2 ).

### A4 Performance characteristics

Using the worst case definition, performance characteristics were similar whether analysed by sample (or patient): sensitivity 0.19 (0.19), specificity 0.99 (0.99), PPV 0.93 (0.93), NPV 0.78 (0.78), and accuracy 0.79 (0.79). The AUROC was 0.59 (0.54 to 0.64) by patient using the worst case definition.

### A5 Univariate and multivariate analyses of Gram stain

For multivariate analyses using the worst case definition, results were similar compared to those using the best case definition, although age was not significantly associated with subsequent septic arthritis by univariate analysis. Hospital site was significantly associated with septic arthritis in both univariate and multivariate analyses (Hull 20 %, other sites 38 % septic arthritis, p<0.001 univariate and multivariate), as was septic arthritis being more likely to be a non-knee joint ( p=0.046 ). A multivariate model without hospital site improved performance of the model and again found no statistical association between the joint involved and septic arthritis. Again, these analyses did not change our overall results or conclusions.

**Table A1:** Definitions of performance characteristics used.

Performance characteristic	Definition and equation
Sensitivity	How good Gram stain is at identifying patients with septic
	arthritis according to our definition (culture or PCR positive)
	True positives (Gram stain positive)
	All with septic arthritis (culture or PCR positive)
Specificity	How good Gram stain is at identifying patients who do not
	have septic arthritis according to our definition (culture or PCR
	positive)
	True negatives (i.e. Gram-stain-negative)
	All who do not have septic arthritis (culture- or PCR-negative)
Negative predictive value (NPV)	In the event of a negative test (Gram stain), the probability that
	the patient does not have septic arthritis
	True negatives
	All negatives
False positives	Samples or patients with a positive Gram stain when septic
	arthritis is not present according to our definition (i.e. a
	positive culture or PCR result)
False negatives	Samples or patients with a negative Gram stain when septic
	arthritis is present according to our definition (i.e. a positive
	culture or PCR result)
Accuracy	The ability of Gram stain to differentiate patients with septic
	arthritis from those without infection correctly
	True positives and true negatives
	All patients or samples
Receiver operating curve	Higher values indicate better diagnostic performance. An
	AUC = 1 indicates perfect performance, whereas an AUC = 0.5
	indicates there is a 50:50 chance Gram stain will correctly
	identify those with septic arthritis.

**Table A2:** Two by two table for calculation of performance characteristics by patient and using the worst case microbiological definition for septic arthritis.

Culture	Gram	Gram	Totals
or PCR	stain	stain	
positive	positive	negative	
(i.e. septic			
arthritis)			
Yes	38	163	201
No	3	584	587
Totals	41	747	788
